# Supplementary figures and images for: Optical Genome Mapping Enables Detection and Accurate Sizing of RFC1 Repeat Expansions
Source: Biomolecules. 2023 Oct 19;13(10):1546. doi: 10.3390/biom13101546 (PMC10605474; doi:10.3390/biom13101546)

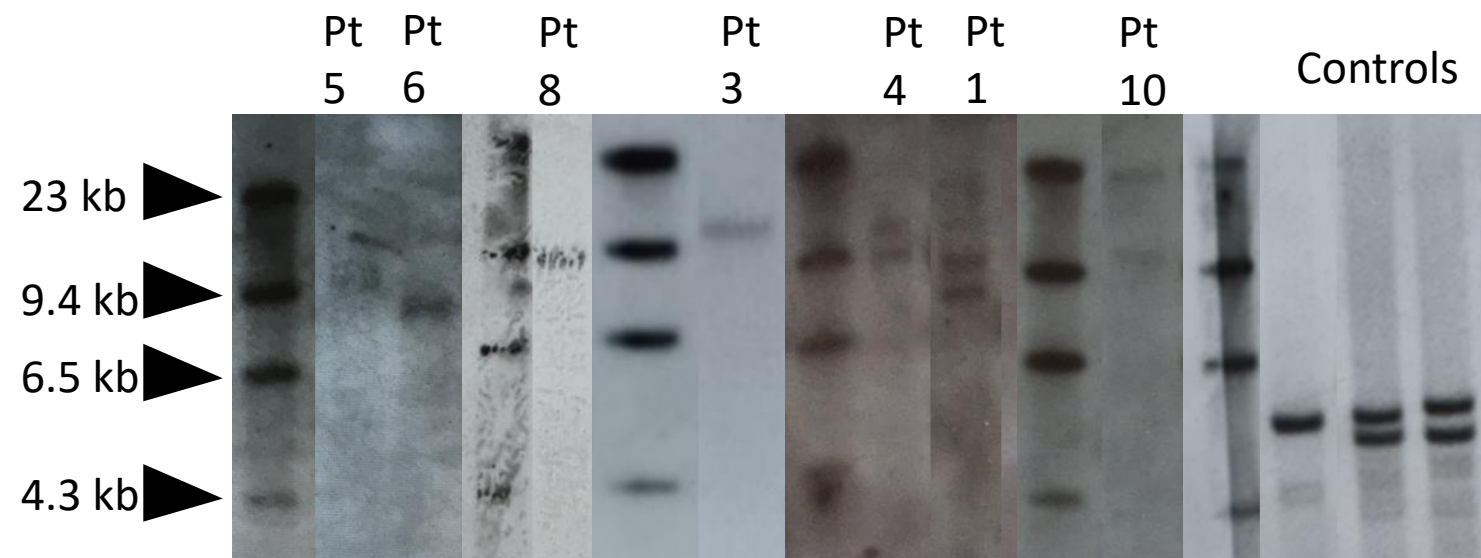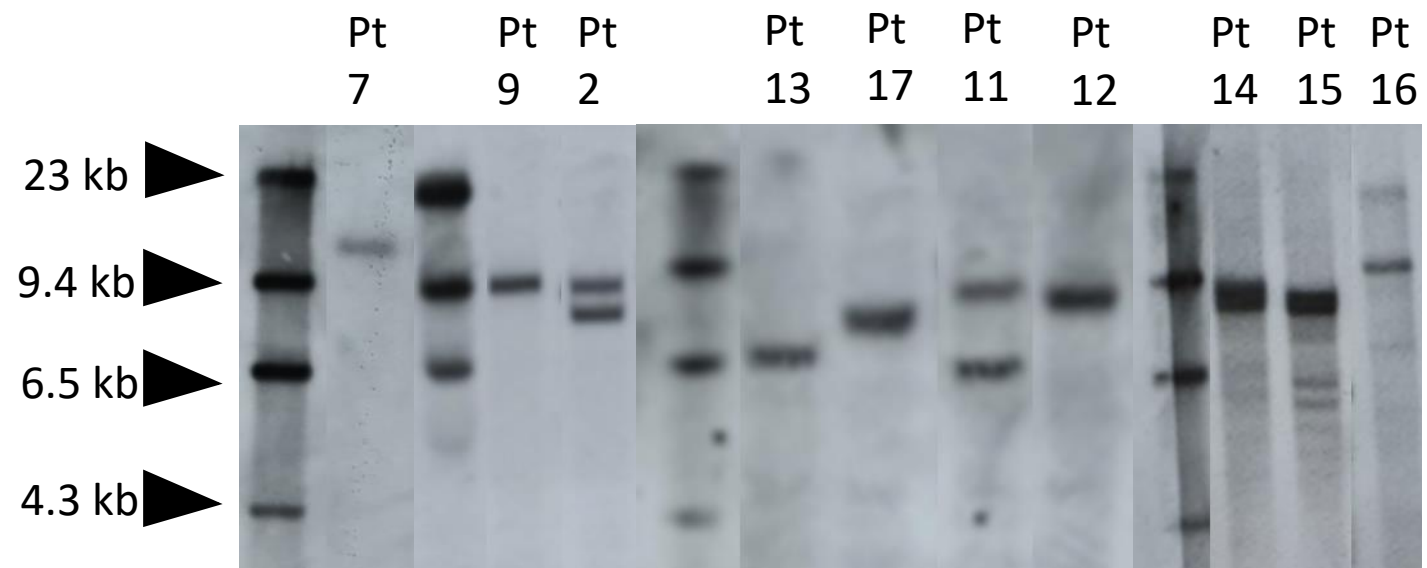

Supplement: Supplementary file 1 [file biomolecules-13-01546-s001.zip › Figure S1.pdf]
